# Impairment in the behavioral control of body sway, gaze shift, and mental workload in Parkinson’s disease

**DOI:** 10.1038/s41598-025-12878-8

**Published:** 2025-08-19

**Authors:** Yann-Romain Kechabia, Luc Defebvre, Arnaud Delval, Cédrick T. Bonnet

**Affiliations:** 1 Univ. Lille, CNRS, UMR 9193 - SCALab-Sciences Cognitives et Sciences Affectives, F-59000 Lille, France; 2 CHU Lille, Hôpital Salengro, F-59000 Lille, France; 3 Faculté de médecine, Unité INSERM 1172, Université de Lille, F-59000 Lille, France; 4 Service de neurologie et pathologie du mouvement, centre expert Parkinson, Hôpital Salengro, CHU, F-59000 Lille, France; 5 Service de neurophysiologie clinique, Hôpital Salengro, CHU, F-59000 Lille, France

**Keywords:** Human behaviour, Neurological disorders, Parkinson's disease, Diseases of the nervous system, Motor control

## Abstract

We tested Parkinson’s disease (PD)-related impairments in the relationship between gaze shifts, body sway and mental workload while performing visual tasks in the standing position. Nineteen on-drug PD patients (Hoehn and Yahr I-II; MDS-UPDRS score part III: 23.37 ± 2.79) and twenty age-matched controls explored large images (visual angle: 100°) and performed a search task (location of targets within images) as well as a free-viewing (control) task. To collect kinematic data, all participants wore body markers (lower back, upper back and head) and an eye tracker. PD patients showed a higher amplitude in gaze shifts and body sway than age-matched controls. The adaptation of gaze shift and body sway velocity from free-viewing to searching was smaller in PD patients. The mental workload (NASA-TLX score) was a significant covariate in all participants. Furthermore, the MDS-UPDRS score was a significant covariate in the shared variance between body (lower back, upper back and head) and eye movement, thus showing a relation between this clinical variable and impairment at the behavioral level. Our results indicate impaired behavioral synergic, i.e. complementary, control between vision, posture and mental workload in PD patients. With a view to restoring synergic functional control, rehabilitation programs should train the three systems together simultaneously.

## Introduction

Parkinson’s disease (PD) is a common neurodegenerative disorder^1^ that induces postural^2^ visual^3,4^ and attentional impairments^5^. In postural control, previous research has shown two main types of synergic behavioral impairment in PD, i.e., muscular synergic impairments^6–8^ and behavioral synergic impairments^9^. The term ‘synergy’ here literally refers to ‘working together’ and therefore refers to the interaction and complementarity between systems.

In the muscular synergic approach, researchers focus on how various leg muscles work together to maintain equilibrium in the standing position. In their studies, Mileti et al.^7^ and Ricotta and Latash^8^ showed that PD patients exhibit synergic impairments in the control of postural muscles. Furthermore, Falaki et al.^6^ found that PD induces an impairment in co-activation and synergic control between various muscle groups required to control balance in the upright stance. Impairments in muscle synergy led PD patients to have a lower ability to coordinate their motor system to control their balance. In the behavioral synergic approach, researchers focus on how various systems, here the postural, visual and attentional systems, work together to perform various types of tasks^10,11^. In Bonnet et al.^9^we showed a PD-related synergic behavioral impairment between the visual and postural systems as PD patients only exhibited significant positive correlations between gaze shift and body sway in a visual search task (to locate specific objects in a room)^9^. In this study, positive correlations were discussed as showing a destabilization effect as the further away the eyes moved, the more PD patients swayed in searching. We should mention that healthy young adults usually show a stabilizing effect between gaze shifts and body control in a search task, referred to as functional synergic control^10,12,13^). Complementarily to our study dealing with relations between vision and posture, D’Ostilio et al.^14^ also evidenced PD-related correlated impairments in gaze shift and body sway. In an anti-saccade task, these investigators showed greater latency and more anti-saccade errors in PD patients than in age-matched controls^14^.

In the behavioral synergic approach, both D’Ostilio et al.^14^ and Bonnet et al.^9^ did not investigate PD-related impairments in the combined relationship between gaze shift, body sway, and attention. However, it may be important to consider how PD-related impairment in attention could influence PD-related impairment in eye and/or body movement as PD patients are known to engage more attentional resources than age-matched controls when performing any visual task^12^. Moreover, PD patients may exhibit greater impairments at a global synergic level, i.e. at the level of interaction between gaze shift, body sway and attention, than at individual levels (either posture, vision, or attention alone) as discussed by two published studies^9,15^.

In the present study, PD patients and age-matched controls performed a visual search task (e.g., locate specific objects in a room) and a control free-viewing task in the standing position. We expected to replicate PD-related synergic impairments between gaze shift and body sway as observed in Bonnet et al.^9^. As a novel finding, we expected to find PD-related synergic impairments between gaze shift, body control, and attention. Furthermore, postural instability and visual impairments are frequently related to the severity of the Movements Disorders Society-Unified Parkinson’s Disease Rating Scale (MDS-UPDRS) part III^16^. Therefore, we expected greater synergic impairments to be related to the MDS-UPDRS score.

## Results

### Velocity of gaze shift and postural sway

**Fig. 1:**
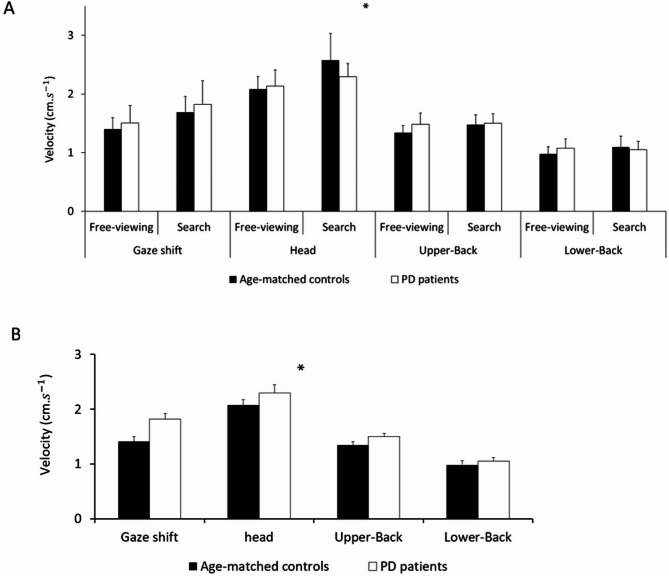
Results of the MANCOVA (Tasks (free-viewing vs. search) by Groups (PD patients vs. age-matched controls) for the mean velocity of postural sway (head, upper back and lower back sway) and gaze shift velocity (cm.s^−1^) on the mediolateral/left-right direction. This figure shows the significant interaction between group (PD patient/age-matched control) and task (search/free-viewing). It also shows the significant main effect of Groups. The significant Tasks by Groups interaction effect and the significant main effect of Groups are shown with a star (*; *p* < 0.05).

The MANCOVA showed a significant task×group interaction effect for the velocities of gaze shift and postural sway (for the head, upper back, and lower back) (*F*_5,78_=6.39, *P* < 0.001; Fig. 1A). Furthermore, the MANCOVA showed a main effect of group (*F*_5,78_=4.17, *P* = 0.002; Fig. 1B). In fact, the variations in the velocities of gaze shift and postural sway were significantly lower in the PD patients than in the age-matched controls in all tasks (Fig. 1A).

To better understand the significant task×group interaction effect (Fig. 1A), post-hoc Newman-Keuls analyses showed that the PD patients moved their gaze, upper back, and lower back (all together as a unit) significantly faster and their head significantly slower than the age-matched controls (*p* < 0.05; Fig. 1A). Another way to understand this result is that the PD patients increased their variance less, and thus their shared variance, than the age-matched controls in body movement (head, upper back, lower-back) and gaze shift from free-viewing to searching. This result is depicted in Fig. 1A by comparing the difference in amplitude between both white barres for each sensor in the PD patients and between both black barres for the same sensors in the age-matched controls. Furthermore, the NASA-TLX global score was a significant covariate in this MANCOVA (*F*_5,78_=5.20, *P* < 0.001). This means that the subjective mental workload for all participants was significantly related to the association between gaze shift and postural control. Further analyses on the NASA-TLX global score are performed in the ‘Complementary analyses’ sub-section to better understand this last finding.

### Variability of gaze shift and postural sway

**Fig. 2:**
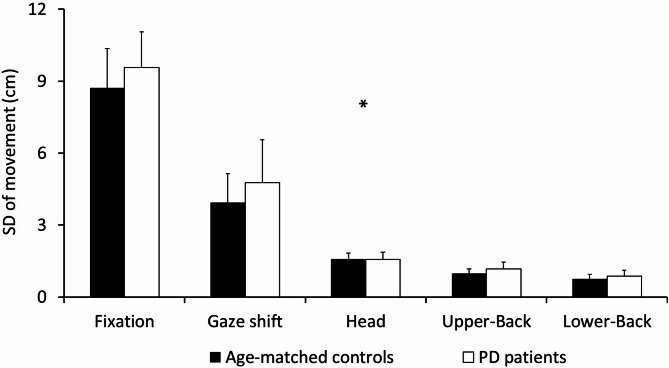
Significant differences between the two groups (PD patients vs. age-matched controls) for the mean postural sway (head, upper back and lower back sway) and mean gaze shift on the mediolateral/left-right direction (cm). The significant Tasks by Groups interaction effect and the significant main effect of Groups are shown with a star (*; *p* < 0.05).

The MANCOVA showed a significant main effect of group in the relationship between the SD amplitude of gaze shift and postural sway (for the head, upper back, and lower back) (*F*_6,80_=3.75, *P* = 0.002, Fig. 2). Post-hoc Newman-Keuls analyses showed that the PD patients moved their gaze and their body lower back, upper back, and head (all together as a unit) more in (SD) amplitude in both the ML axis and LR directions than the age-matched controls (Fig. 2). Furthermore, the mean NASA-TLX global score was a significant covariate in this MANCOVA (*F*_6,80_=3.84, *P <* 0.001). As previously observed, it means that the subjective mental workload for a given task was significantly related to the association between gaze shift and postural control (see ‘complementary analyses’ to further understand this last finding).

### Influence of the MDS-UPDRS III

Two MANOVAs (one for the velocity and one for the variability) were performed to control the effects of a clinical variable (MDS-UPDRS III score) on the behavioral synergy. The results showed that a more severe MDS-UPDRS III (cf. Table 2) was associated with a lower degree of synergic control in velocity (*F*_12,79_=3.42, *P* < 0.001) and in the variability of gaze shift and postural sway (*F*_13,94_=3.49, *P* = 0.001).

### Complementary analyses

#### Changes in subjective mental workload

As the NASA-TLX global score was a covariate in the two MANCOVAs, we further investigated the evolution of this score between the two tasks and also between the two groups by using a posteriori repeated measures ANOVA with two factors (Group and Tasks). Consistent with results from the MANCOVA, the NASA-TLX global score was higher in the search task (7.92 ± 3.71) than in the free-viewing task (6.14 ± 3.33) for all participants (*F*_1,36_=12.92, *P* < 0.001). However, the main effect of group and the group by task interaction effects were not significant (*F*_*s*1,36_<0.98, *P* > 0.05).

#### Task performance in the search task

To verify compliance with the instructions, one ANOVA was performed to study the number of target objects found by each participant in each trial of the search task. In this analysis the PD patients (mean ± SD: 7.21 ± 2.36) and the age-matched controls (mean ± SD: 8.18 ± 2.34) located the same number of targets (*F*_1,37_=1.65, *P* > 0.05). This result provides a relevant finding to adequately compare differences in motor control (visual and postural) between the two groups as all participants performed the search task in similar ways.

#### Angular head displacement in the Yaw direction

For complementary analyses, we analyzed the velocity of angular head displacement in the left-right direction to illustrate how fast the participants rotated their head in both free-viewing and search tasks to control for this potential confounding variable. The results showed that the PD patients rotated their head significantly more slowly (PD patients: 1.94 ± 0.55 °/s) than the age-matched controls (2.25 ± 0.56 °/s; *F*_1,74_=5.88, *P* = 0.0018). This analysis showed that (i) our participants rotated their head extremely slowly to explore images and that (ii) PD patients rotated their head even more slowly than age-matched controls.

## Discussion

Our objective was to study PD-related impairments in synergic, i.e. complementary, control between gaze shift, postural sway and attentional workload in visual tasks performed in the standing position. We found that in the visual search task, the PD patients were not able to adjust the velocity and amplitude of their gaze shift and postural sway as much as the age-matched controls. In both the PD patients and age-matched controls, the amount of shared variance between gaze shifts and postural control was significantly related to the subjective mental workload. Our study also showed significant positive relationships between the severity of PD (the UPDRS part III score) and the impairments in behavioral synergic control.

### PD-related impairments in gaze shifts and postural sway

Our results validated our original hypothesis that we would find PD-related impairments in synergic control between gaze shift and postural sway. Indeed, we found a significant group by task interaction effect in the first MANCOVA (testing the velocity of gaze shift and postural sway). This analysis showed that the PD patients changed their eye and body velocity less than the age-matched controls to perform and succeed in the search task (vs. the free-viewing task). As described in the Results section, the PD patients showed less change in eye and body movement velocity than the age-matched controls between our two task (free-viewing/searching; Fig. 1A). It is also important to understand that the PD patients exhibited lower velocity in gaze shifts and postural sway than the age-matched controls (significant main effect of group; Fig. 1B). The PD patients thus showed a lack in behavioral adjustment from free-viewing to searching to succeed in the search task. Additionally, the second MANCOVA (testing the variability of gaze shift and postural sway) showed that the PD patients moved their eye and body more than age-matched controls in all tasks (Fig. 2). This result thus validated our previous finding. Firstly, PD patients exhibited greater body and eye movements (Fig. 2). Secondly, PD patients demonstrated a reduced ability to produce complementary movements compared to age-matched controls (Fig. 1A). Overall, our results confirmed the view that PD patients exhibit an “impaired control of action stability”^17–19^ also called “maladaptive adjustments”^19^.

The results in the present study confirmed the PD-related impairments in synergic control between gaze shifts and postural control as showed in our previous study using the same set-up^9^. The results in the present study were also complementary and slightly different than in our prevous study^9^. In Bonnet et al.^9^we used Pearson’s correlation analyses to assess synergic impairments in PD and found no significant negative correlations in PD patients, typically indicating stabilizing control. Instead, PD patients exhibited significant positive correlations, suggesting destabilizing control. Therefore, based on Pearson’s correlation analyses, we previously described an impairment in behavioral synergic control in PD⁹. PD patients exhibited a complete inability to coordinate gaze shifts and postural sway. In the present study, using MANOVAs, we found evidence of synergic impairments as well. This time, PD patients exhibited a reduced ability to engage synergic control rather than a complete inability to coordinate gaze shifts and postural sway. We believe this difference is due to the methods to analyze the data. Indeed, on one hand, pairwise correlation analyses are not sensitive enough to capture subtle variations, as they only revealed the presence or absence of significant relationships. On the other hand, MANOVAs examine variations in movements (here head, upper back, lower back, and gaze) simultaneously. These analyses enabled us to detect significant differences in oculomotor-postural behavior between the two groups. Our results therefore add to the literature reports. We assume that our new results using MANOVAs align more closely with previous findings in the literature and better reflect PD-related impairments in synergic control. Indeed, studies investigating muscular synergy in PD have shown that patients exhibit greater variability in muscle activation compared to age-matched controls, both in upper body muscles^20^ and lower body muscles^21^. These published findings showed a reduced ability to coordinate muscle activity for balance control in PD. They also suggested, as we do in our study, that PD patients were less able to use muscle synergies to maintain an upright stance^7,17^, rather than being entirely unable to do so.

### PD-related impairments in gaze shifts, postural sway and subjective mental workload

Our results extend our previous findings^9^ by showing that behavioral synergic impairments were also linked to the subjective mental workload. Indeed, in both MANCOVAs, subjective mental workload—as measured by the global NASA-TLX scores—emerged as a significant covariate. In other words, for both PD patients and age-matched controls, the degree of shared variance between gaze shifts and postural control was significantly associated with perceived mental workload.

On one hand, our results on subjective mental workload (see MANCOVAs and complementary results) showed that PD patients at Hoehn and Yahr stages II and III, and while on medication, were still able to use a goal-directed mode of control. This mode of control is cognitively demanding. Despite their disease, these patients performed the visual search task as effectively as age-matched controls using this strategy. This finding is in line with previous research. It is well established that PD patients tend to rely more on goal-directed control than on automatic control. This shift is considered a compensatory mechanism caused by the disease itself^19,22–24^. On the other hand, our study showed that PD patients were less able than age-matched controls to coordinate gaze shifts and postural control (see Fig. 1A and B, and 2). This difficulty in performing synergic behavior was observed despite both groups reporting similar levels of subjective mental workload. Two interpretations could explain our results. A first explanation may be that PD patients exhibited difficulties coordinating eye movements, posture, and attention because of problems at the behavioral level and not at the attentional level. In line with this first explanation, PD patients may have adopted an ‘avoidance behavior’ to protect themselves against disequilibrium^25^. They might have shown PD-related lower amplitude and velocity of eye and body movements (cf. Figures 1 and 2) in order to specifically control their postural stability^26^ in detriment of their functional ability to interact with their environment. A second explanation is that, even though PD patients and age-matched controls reported the same level of attentional effort, this was not enough for PD patients to coordinate eye movements and postural sway as effectively as the controls. In line with this second explanation, the PD patients are known to exaggerate their use of attentional resources to compensate for their impairment in attention and in executive functions^14,19,27–29^. Future research is required to know which interpretation is more accurate.

The results of the present study are in line with those of Ewenczyk et al.^14^ and bring complementary insights. In their study, PD patients and age-matched controls performed an anti-saccade task inside an fMRI scanner. They also completed a quiet stance trial. Based on the recorded postural data, the PD group was divided into two subgroups: one stable and one unstable. Ewenczyk et al.^14^ found impairments in the link between gaze shifts and postural sway in PD. The unstable group showed longer reaction times and made more anti-saccade errors than the controls. They also had reduced functional connectivity between the frontal eye fields and the pedunculopontine nucleus. Ewenczyk et al.¹⁵ did not directly assess synergic impairments. However, like us, they reported related difficulties involving posture, eye movements, and cognitive effort in PD patients.

Ewenczyk et al.^14^ also studied the relations between visual, postural and attentional impairments and neurophysiological variables. Their analyses showed that the group of less stable PD patients was impaired in the relation between posture, vision and neural activation between the frontal eye field and the PPN compared to the group of more stable PD patients. In line with these findings, we showed that the severity of PD (MDS-UPDRS III score) was proportional to the impairment in synergic gaze, posture and subjective mental workload. Future studies need to further investigate the link between clinical variables and synergic impairments as they seem to be quite well related (Table 1).

**Table 1:** Demographical data of the 39 participants. Table 1 shows the number of participants in both the group of patients with Parkinson’s Disease (PD patients) and age-matched controls, the gender, the mean age (in years), the mean height (in meters), the mean bodyweight (in Kilograms), the MOCA score (Montréal Cognitive Assessment score), the mean disease duration, the MDS UPDRS part III (Motor Experiences of Daily Living-Unified Parkinson’s Disease Rating Scale part III), the levodopa mean equivalent dosage (in mg per day), the Hoehn and Yahr score and the axial rigidity score (computed with values from the MDS UPDRS part III items 18, 22, 27, 28, 29, and 30^49^).”.

	PD patients	Age-matched controls
n	19	20
Gender	16 males/3 females	15 males/5 females
Mean age*	58.47 ± 2.31	61.56 ± 1.91
Mean height*	1.76 m ± 0.02	1.72 m ± 0.02
Mean bodyweight*	77.89 kg ± 3.96	83.11 kg ± 3.37
MOCA score	27.68 ± 1.25	
Disease duration	7.36 ± 4.12 years	
MDS UPDRS part III	23.37±2.79	
levodopa mean equivalent dosage	658.6 ±239.04 mg/day	
Hoehn & Yahr score	2.16±0.37 (range: 1‒3)	
Axial rigidity**	1.74 ± 0.36	

* differences between PD patients and Age-matched controls were not significant (p > 0.5).

**calculated by summing UPDRS III items 18, 22, 27, 28, 29, and 30.

### The neurophysiological basis of PD-related impairments in synergic control

Two overall neurophysiological bases for PD-related impairments in synergic control can be discussed. Several studies have suggested that the basal ganglia are involved in gaze shifts^30,31^ in the neural pathways for gaze shift and postural sway^32^ and in attention^30,31^. In fact, some studies in muscle synergy have associated neurophysiological damage to the basal ganglia in PD with impairments in postural control^7,8,33^. The basal ganglia also appear to be involved in the switch from one control mode to another^19,23^. Consistent with these published manuscripts, the basal ganglia may well explain PD-related impairments in the behavioral synergic control found in our study (Figs. 1A and B and 2). Moreover, Lewis et al.^32^ suggested that damage to the PPN observed in most PD patients can explain isolated PD-related impairments in gaze shifts, postural sway, and/or attentional resources. In fact, the PPN is known to be involved in postural muscle tone^30,32^ and it is a relay for anticipatory postural adjustments^30,34^. When the PPN is damaged, PD patients show a greater postural sway velocity^35^. The PPN is also involved in the preparation and initiation of saccades^14,36^. This nucleus receives inputs from the supplementary motor area (SMA) and the frontal eye fields. The SMA is involved in generating voluntary saccades, while the frontal eye fields are responsible for planning these eye movements^14^. The PPN also plays a role in attention, due to its connections with the medial prefrontal cortex^34,37^. Ewenczyk et al.^14^ showed that unstable PD patients had reduced connectivity between the SMA and the PPN, as well as between the frontal eye fields and the PPN. Both Ewenczyk et al.^37^ and Gallea et al.^37^ suggested that the PPN acts as an integrative nucleus. It is involved in goal-directed movements, action selection, and rapid movement-related decisions. As we did not record any brain activity, our results cannot be used to decide which of the two hypotheses better explains the synergic impairments observed in PD. Future studies will need to clarify the respective roles of the basal ganglia and the PPN in muscular and behavioral synergy deficits in PD. Recent research suggests that the basal ganglia may act more as a modulator than as a trigger for actions³⁴. In other words, the basal ganglia may adjust the strength of activation or inhibition, rather than directly controlling whether a behavior is initiated or blocked.

The vestibular system is known to be involved in synergic control of eye and head movements^38^. However, the vestibular system may not play a major role in explaining our results. In fact, the PD patients were at an early stage of their disease, with no freezing of gait or severe axial impairment (mostly Hoehn and Yahr stage II, see Table 2). Accordingly, previous investigators found no relation between disease progression and impairment of the vestibular system^39,40^. Moreover, in our study, PD patients turned their head extremely slowly to explore images (see complementary results). In fact, the vestibular system could still have a role in the synergies between visual, postural, and attentional systems if individuals were turning their head and/or body quickly. Future studies with quick head rotations should test this possibility.

**Table 2:** Hoehn and Yahr stage and MDS UPDRS III scores of the 19 PD patients.

PD patients	Hoehn and Yahr stage	MDS-UPODRS III
1	2	29
2	2	8
3	2	38
4	3	51
5	2	19
6	2	8
7	2	25
8	3	40
9	2	27
10	2	33
11	2	10
12	2	10
13	2	24
14	2	10
15	3	10
16	2	22
17	2	30
18	2	23
19	2	27

## Limitations

The present study has several limitations. Firstly, the PD group included only patients at relatively mild stages of the disease (Hoehn and Yahr stages I–II), and all were tested in the on-medication state. Hence, we cannot generalize our findings to PD patients at a more advanced stage of their disease or in off-medication conditions. However, previous research suggests that medication does not improve synergic control, or only improves it slightly^7,20,21^. Secondly, the cross-sectional design of our study prevents any conclusions about the progression of synergic impairments over time. Future longitudinal studies will be necessary to assess the evolution of these impairments along time. Thirdly, our assessment of attentional involvement was indirect, as we relied on the NASA-TLX to measure subjective mental workload. Nonetheless, this tool has been already used in studies involving PD patients^33,41,42^ and has shown significant correlations with objective measures of attentional load in both PD patients^43,44^ and healthy adults^45^ Fourthly, we did not examine the potential impact of axial rigidity or other axial symptoms on synergic impairment, as we had no a priori hypothesis and wanted to avoid increasing the risk of type I error. However, this perspective remains a promising area for future studies involving larger and more diverse samples. Fifthly, we did not investigate visual, postural, or attentional impairments individually. Instead, we focused on integrated (i.e., synergic) impairments by examining shared variance between these systems. We did so to be able to test our primary aim: to explore multisystem coordination rather than isolated deficits.

## Conclusion and perspectives

As in previous studies^12,14^, our results showed that Parkinson’s disease impairs the behavioral interaction between postural control, ocular control, and subjective mental workload. Practically, this suggests that PD patients may struggle to adapt their posture and gaze to succeed in visually demanding tasks. We recommend that PD patients, even from Hoehn and Yahr stage II, engage in targeted training involving visual tasks and engaging balance, eye movement and attention all together. One practical and accessible approach could involve video games played in a standing position, where task performance requires quick coordination between body movements and gaze (e.g., catching visual objects, hitting moving targets). Previous studies have already shown promising effects of video game–based interventions in PD patients^46,47^. Looking forward, future research should include longitudinal studies to examine how synergic impairments progress over time and how they respond to targeted interventions. Intervention trials focused on multisystem rehabilitation, addressing vision, posture, and attention together, would be especially valuable to test the clinical impact of integrated training approaches.

## Materials and methods

### Participants

Nineteen PD patients and 20 healthy age-matched controls participated in the study. This sample size was considered to be sufficient. Indeed, in a one-tailed test in G*Power (G*Power software, version 3.1.9.2; Düsseldorf, Germany), an estimated effect size (f) of 0.95 (as seen in our previous study^9^ an alpha risk of 0.05, a power of 0.8, and a phi correlation (H0) of 0 yielded a minimum sample size of 16 participants per group. The PD patients were recruited during consultations in the Neurology Department at Lille University Medical Center (Lille) and the age-matched controls were recruited by the University Medical Center’s Clinical Investigation Center.

There were no significant differences in physical and demographic characteristics between the PD patients and the age-matched controls (Tables 1 and 2). In fact, the age, bodyweight and height were not significantly different between both groups as shown by three t-tests (t(37) = −1.23, t(37) = −1.18; t(37) = 1.34; all p_s_>0.05). All the participants met the inclusion criteria, i.e. they had normal or corrected vision and thus could clearly see and explore the experimental images. None of the participants met any of the non-inclusion criteria: none of the participants had a history of neurological disease (except for PD in the patient group), musculoskeletal disease, vestibular problems, recurrent dizziness, dementia, motor fluctuations, subclinical dyskinesia, or any falls in the previous six months. All the PD patients had a Montreal Cognitive Assessment (MoCA) score greater than or equal to 25^48^. The mean MoCA score for the PD patients was 27.68 ± 1.25. The PD patients were tested in the “on-drug” condition and were stable for their motor evaluation (no fluctuations). The averages for disease duration, mean MDS-UPDRS score part III, Hoehn and Yahr stage, axial score^49^ and levodopa equivalent dosage per day are shown in Tables 1 and 2. The study was performed in accordance with the tenets of the Declaration of Helsinki and was approved by the French Ethical Committee (n°2014-74) Nord Ouest IV. All the PD patients and age-matched controls gave their written, informed consent to participate.

### Experimental tasks

#### Apparatus

**Fig. 3:**
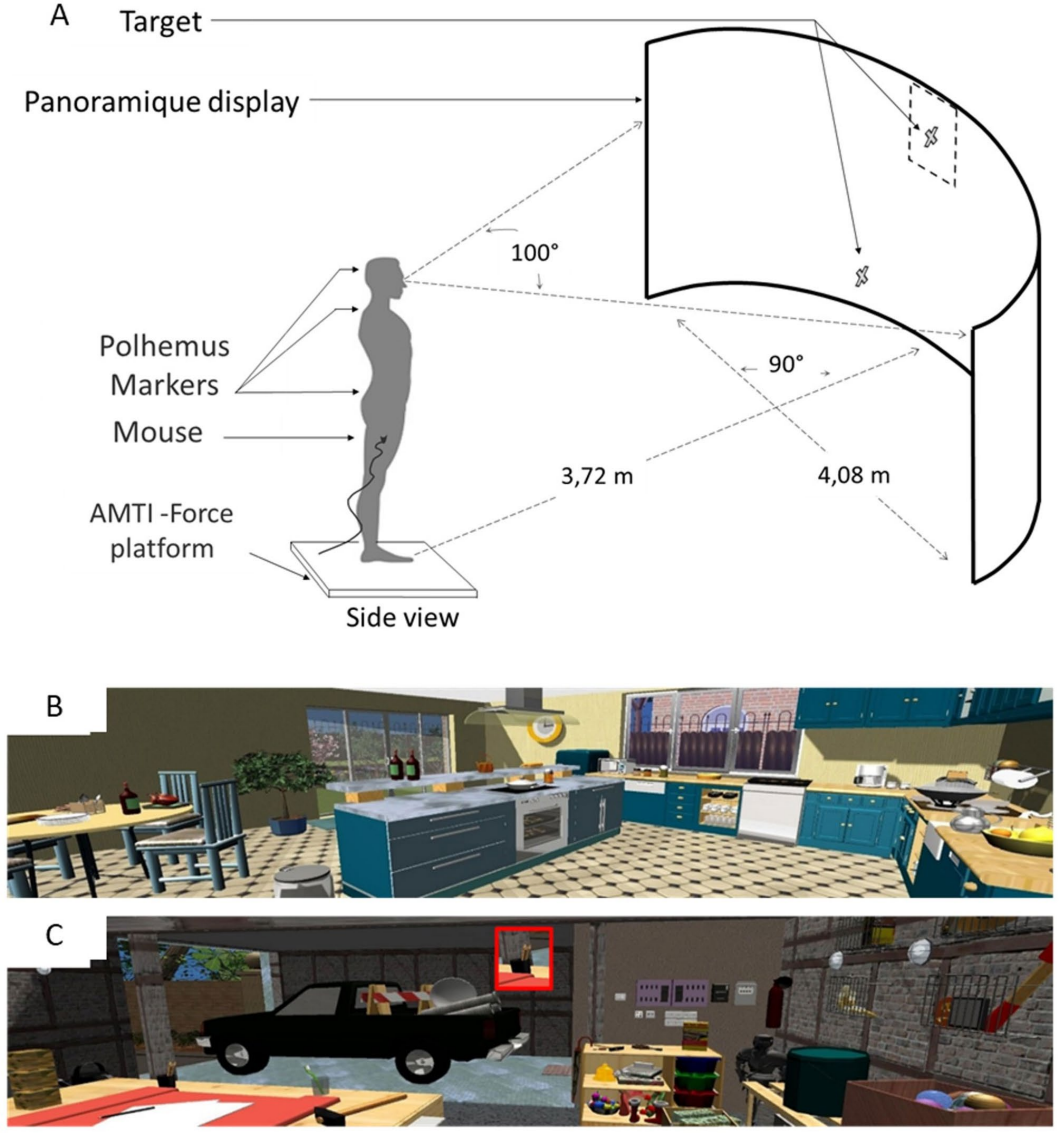
Experimental set up. (**A**) The participants stood in front of a semi-circular panoramic display and could see the image with a visual angle of 100°. The participants wore an onboard SMI oculometer and three Polhemus markers attached at the level of the head, upper back and lower back. The experimental images (designed in an ecological way) were projected on the panoramic screen. The participant was positioned in front of the display. Two experimental images are illustrated in B and C. (**B**) In the free-viewing task, there was no red square and no target to be found. The participants simply explored the image with no specific goal. (**C**) In the search task, a red square was shown at the top and middle of the image to show a target to be found within the image. Once the participants had located the target within the image, they had to look at it and click on the mouse (to validate their finding) and to search to locate a new target.

The experimental images (rooms in houses, see Fig. 3B and C) were projected on a full panoramic display (diameter: 2.04 m; height: 2 m; visual angle: 100°; Fig. 3A). Three Polhemus markers (Liberty 240/8–8 System, Polhemus, Colchester, VT) were positioned on the lower back, upper back and head with a sampling frequency of 240 Hz (Fig. 3A). The markers’ dimensions were width: 2.28 cm, depth: 2.79 cm and height: 1.52 cm. An eye tracker (SensoMotoric Instruments, Teltow, Germany) recorded movements of the right eye (Fig. 3A) with a frequency of 50 Hz. During trials, the participants held a computer mouse in their preferred hand and pressed it against their thigh (Fig. 3A). A custom MATLAB script (version 7.10.0, MathWorks Inc., Natick, MA) was used to synchronize the recordings of the three devices. A French version of the National Aeronautics and Space Administration Task Load Index (NASA-TLX) questionnaire^45,50^ was used to quantify the subjective mental workload in each task. The NASA-TLX questionnaire has already been used in studies with PD patients^33,41–44^.

#### Tasks and instructions

The participants performed a non-precise (free-viewing) and a precise (search) task (Fig. 3B and C, respectively). There were five trials per task, and each trial lasted 48 s. In the free-viewing task, the participants visually explored the experimental image as they liked (Fig. 3B). In the search task, the participants had to locate as many targets as possible in the image and click on the mouse when they found a target. Each target was presented in a red square centered at the top of the screen (Fig. 3C). The click validated their finding and automatically changed the target in the red square centered at the top of the screen (Fig. 3C). After each click, the target was new as we prepared 30 targets for each image. They could freely turn their eyes, head, shoulders and more generally body parts as they liked during trials but they had to keep standing on their two feet. In both conditions, participants held the mouse in their preferred hand but only used it in the search task.

#### Procedure

Once the participants arrived at the hospital Roger Salengro in Lille, they read the information letter and gave their written informed consent. Then, they were examined by an experienced neurologist (LD or AD), who verified compliance with the inclusion and non-inclusion criteria. After this initial clinical investigation, the participants were guided to the experimental room. The investigator attached the various Polhemus magnetic markers to the occiput (head marker, on the headset), at the seventh cervical vertebra (upper back marker), and at the fifth lumbar vertebra (lower-back marker, on a chest belt), as well as the head-mounted SMI eye tracker (SensoMotoric Instruments, Teltow, Germany).

During the study, the participants stood upright in front of the panoramic display (Fig. 3A), in a standardized position indicated by marks on the ground. The distance between the feet was defined and kept constant in agreement with McIlroy & Maki’s normative data (17 cm, 14°^51^). Once the participants were prepared, they were then given task instructions before performing two preliminary training trials. The investigator turned the light off, calibrated the Polhemus markers and the eye tracker. The participants performed five trials in one task (free-viewing or search) before performing the other task. We chose to perform five trials per task to increase the validity of each of our dependent variables as the participants could move/rotate their body segments quite differently from one trial to another, since they could move them as they wish to succeed in the task The order of the task, the 5 images per task, and the displayed targets were all randomized and the participants could never see any image and any target twice or more. The participant was invited to sit and rest after each task (after five successive trials). They filled out the NASA-TLX questionnaire when seated. During the study, the experimenter checked that the participants performed the task correctly in looking at the video of the eye tracker continuously showing where the participants looked at.

#### Dependent variables

The image of the target objects to be found was located in the red square at the top of the panoramic screen. This choice could have led the participants to exaggerate gaze shifts in the top-down direction and body sway on the anteroposterior axis. Furthermore, eye and body movements were largely greater, and therefore more variable, on the mediolateral (ML) axis than on the anteroposterior axis, respectively with visual angles of 100° and 23°. For these reasons, we studied only body movements on the ML axis and gaze shift in the left-right (LR) direction (we did not study them on the AP axis and up-down direction, respectively).

*Body sway*. Conventional dependent variables (positional standard deviation and mean velocity) were used to study movements of the lower back, upper back, and head^52^.

*Gaze shifts*. For gaze shift time-series, we also used the standard deviation and mean velocity in the LR direction. LR was preferred to ML since in the literature on vision, the eyes are usually illustrated to move in the LR direction. To analyze the characteristics of fixation, we studied the eye’s fixation position in all fixations performed during trials. For analyses, we used the positional standard deviation of all fixations. We did not compute or analyze the mean velocity of fixations, as this variable was irrelevant.

*NASA-TLX*. The subjective mental workload was computed with the NASA-TLX global score for each task. This variable was relevant in our study, since several studies already evidenced a direct association between the subjective mental workload and objective measures of attentional involvement in patients with early- or mid-stage PD^44,42^. This variable was also relevant as it was previously used in young adults in studies investigating synergic behaviors between gaze shift, body sway, and cognitive workload^10,12,13^.

#### Preparation of data and statistical analyses

The Polhemus data were resampled at 50 Hz. The first three seconds in each trial were not analyzed. As in our previous studies^9–12,53^ we only analyzed trials in which the eye tracker measured more than 80% of the data. In our data, we analyzed 86.02% of the recorded data. Outliers were removed from the datasets, and the standard deviation ratio (the ratio between the short-term standard deviation and the long-term standard deviation) was calculated. Analyses of outliers were performed on each trial. Trials with a standard deviation ratio greater than 3.5 were excluded from our analysis because this value indicates the presence of atypical data^54,55^. In our study, less than 2.5% of data were removed. For all analyses, we worked on an average of the five trials per task for each participant.

We used multivariate analyses of covariance (MANCOVAs) in Rstudio software (Rstudio, Vienna, Austria) to analyze synergic, or complementary, behavior between gaze shifts, body sway (lower back, upper back, head), and subjective mental workload. The two factors in the MANCOVAs were the group (PD patients vs. age-matched controls) and the experimental conditions (free-viewing vs. searching). The role of the subjective mental workload was tested as a covariate in these MANCOVAs. Two MANCOVAs were performed: (A) on the velocity of movement and (B) on the amplitude of movement. These MANCOVAs were novel analyses to discuss synergic impairment, between gaze shift, body sway and the subjective mental workload as we used Pearson’s correlation analyses as in our previous study with PD patients^9^ or in young adults^10,12,13^. We used MANOVAs because these analyses test shared variance, and we can assume that the higher the shared variance is, the higher the synergy – or complementarity or interaction – between system^s is. Furthermore, instead of using partial correlations to control the effect of subjective mental workload on the relationship between eye movement and postural sway as in^10,12,13^we used MANCOVAs with ‘C’ being the controlled subjective mental workload variable. We chose to perform MANCOVAs instead of Pearson’s correlations and partial correlations because MANCOVAs are more powerful. In fact, we only performed two MANCOVAs, combining both groups, both tasks, all markers, and the NASA-TLX variable instead of hundreds of correlations with separated analyses between groups, tasks, and markers, as in our previous studies^9,10,12,13^.

Two additional MANOVAs were performed to control the effects of the clinical variables (UPDRS III score) had on the behavioral synergic control of movement. The first MANOVA was performed with the velocity of movement and the second MANOVA with the variability of movement. A significant interaction between our variable (clinical and movement) indicates that the synergic impairment is associated with the disease progression. We performed our four analyses (two MANCOVAs, two MANOVAs) with a significant level of 0.05. When one of these analyses was significant, post-hoc Newman-Keuls were performed only to determine if there were any significant difference for each of our markers according to our factors (groups and tasks).

## Data Availability

The datasets used and/or analyzed during the current study are available from the corresponding author on reasonable request.
